# Rhythm and conduction disturbances after Tetralogy of Fallot correction

**DOI:** 10.1186/1749-8090-8-S1-O85

**Published:** 2013-09-11

**Authors:** M Chira, DF Ciotlaus

**Affiliations:** 1 Cardiovascular Department, Polisano Hospital, Sibiu, Romania

## Background

Rhythm and conduction disturbances are not uncommon complication after surgical correction of Tetralogy of Fallot. Arrhythmias after surgical correction of congenital heart defects are extensively studied in international literature, but the results are not similar. This study highlights the higher risk of arrhythmias when the TOF surgical correction is delayed.

## Methods

The study is based on a group of 71 pediatric patients operated in Heart Institute between September, 2001 and July, 2006, surgically corrected per primam, without prior palliations. The surgical techniques were: transannular patch (46), infundibular patch (12), infundibular patch + pulmonary patch (5), transatrial + transpulmonary repair (8). The patients were divided into 2 groups, correction under 1 year of age and correction above 1 year of age, for comparative study of results. From the 71 patients group, 58 were followed up at 43 months interval group 1, and 48 months interval group 2, by standard electrocardiography, 24 hours Holter monitoring and echocardiography.

## Results

Major differences between the two groups were recorded: the QRS and QTc duration were significantly different between the 2 groups, right bundle branch block with left anterior hemiblock was noticed especially in patients operated over 1 year of age, ventricular arrhythmias were present especially in the same group of patients operated over 1 year and did not appear in patients with transatrial and transpulmonary repair; furthermore ventricular arrhythmias were especially present in patients with postoperative severe pulmonary regurgitation; the mean QRS duration was intensely significant correlated with the type of arrhythmia.

## Conclusions

Rhythm and conduction disturbances are statistically significant correlated with the age at correction (the greater the age at operation, the bigger the risk for this type of complications), moreover being correlated with the surgical technique, as well.

